# Microfluidic model of monocyte extravasation reveals the role of hemodynamics and subendothelial matrix mechanics in regulating endothelial integrity

**DOI:** 10.1063/5.0061997

**Published:** 2021-09-14

**Authors:** Sandra Pérez-Rodríguez, Stephanie A. Huang, Carlos Borau, José Manuel García-Aznar, William J. Polacheck

**Affiliations:** 1 Multiscale in Mechanical and Biological Engineering, Department of Mechanical Engineering, University of Zaragoza, 50018 Zaragoza, Spain; 2 Aragon Institute of Engineering Research, University of Zaragoza, 50018 Zaragoza, Spain; 3 Joint Department of Biomedical Engineering, University of North Carolina at Chapel Hill and North Carolina State University, Chapel Hill, North Carolina 27599, USA; 4 McAllister Heart Institute, University of North Carolina at Chapel Hill School of Medicine, Chapel Hill, North Carolina 27599, USA; 5 Department of Cell Biology and Physiology, University of North Carolina at Chapel Hill School of Medicine, Chapel Hill, North Carolina 27599, USA

## Abstract

Extravasation of circulating cells is an essential process that governs tissue inflammation and the body's response to pathogenic infection. To initiate anti-inflammatory and phagocytic functions within tissues, immune cells must cross the vascular endothelial barrier from the vessel lumen to the subluminal extracellular matrix. In this work, we present a microfluidic approach that enables the recreation of a three-dimensional, perfused endothelial vessel formed by human endothelial cells embedded within a collagen-rich matrix. Monocytes are introduced into the vessel perfusate, and we investigate the role of luminal flow and collagen concentration on extravasation. In vessels conditioned with the flow, increased monocyte adhesion to the vascular wall was observed, though fewer monocytes extravasated to the collagen hydrogel. Our results suggest that the lower rates of extravasation are due to the increased vessel integrity and reduced permeability of the endothelial monolayer. We further demonstrate that vascular permeability is a function of collagen hydrogel mass concentration, with increased collagen concentrations leading to elevated vascular permeability and increased extravasation. Collectively, our results demonstrate that extravasation of monocytes is highly regulated by the structural integrity of the endothelial monolayer. The microfluidic approach developed here allows for the dissection of the relative contributions of these cues to further understand the key governing processes that regulate circulating cell extravasation and inflammation.

## INTRODUCTION

Extravasation is the process by which cells and proteins traverse the endothelial barrier from the intravascular cannula into surrounding tissue.^1^ During an inflammatory injury or bacterial infection, immune cells extravasate in response to mechanical and chemical stimuli secreted by damaged tissues.^2^ In this work, we focus specifically on monocyte extravasation due to its essential role in homeostasis and the development of innate and adaptive immune responses to pathogens.^3^ While a number of cellular and molecular regulators of extravasation have been identified using conventional *in vitro* approaches,^4^ interpreting these results in the context of the native microenvironment is challenging due to limitations to *in vitro* culture systems, including the lack of physiologic tissue architecture and mechanics. Here, we seek to address these shortcomings through the implementation of a microfluidic multi-cell culture system that more closely mimics the native architecture and mechanics of the microvasculature.

Monocyte extravasation is initiated through the coordination of receptor-mediated interactions between the apical surface of vascular endothelial cells and monocytes. First, immune cells from damaged tissue secrete inflammatory cytokines induce the expression of adhesion molecules in endothelial cells. E- and P-selectin, intercellular adhesion molecule 1 (ICAM-1), and vascular cell adhesion molecule 1 (VCAM-1) have been specifically implicated in monocyte–endothelial interactions.^5^ ICAM-1 and VCAM-1 contribute to monocyte adhesion,^6^ and VCAM-1 reduces rolling speed and enhances firm arrest.^7^ Additionally, monocytes have basal expression of P-selectin Glycoprotein Ligand 1 (PSGL-1), which interacts with endothelial selectins, allowing for the attachment and rolling of monocytes.^8^ Once monocytes attach to the endothelial wall, an increase in endothelial RhoA GTPase-mediated actomyosin contractility in endothelial cells induces intercellular junction disassembly and the formation of transient gaps in the monolayer.^9^ Finally, monocytes generate protrusions to probe the endothelial surface and cross the endothelial barrier.^10,11^

Hemodynamics play a critical role in extravasation through modulating protein expression and signaling in endothelial cells. Fluid shear stress between 2 and 10 dynes/cm^2^ upregulates the expression of E-selectin, P-selectin, ICAM-1, and VCAM-1 on endothelial cells, leading to an increase in monocyte arrest at the endothelial wall.^12–14^ Conversely, endothelial cells exposed to physiological shear stress, in a range between 3 and 5 dynes/cm^2^, suppress RhoA signaling and upregulate Rac1 signaling, resulting in adherens junction assembly and cytoskeletal alignment.^15–17^ Therefore, hemodynamic shear stress imparts competing signals on the extravasation process by increasing monocyte arrest to the endothelium, but strengthening the vascular barrier. Further dissection of these mechanisms necessitates the development of a platform in which vascular endothelial cells and monocytes can be cultured in a hemodynamic environment and observed in real time.

Signals from the basal surface of the endothelium, including the perivascular extracellular matrix, have also been shown to play a role in extravasation. Vascular endothelial cells are highly sensitive to the stiffness of the underlying matrix,^18^ and pathologically increased matrix stiffness drives increases in vascular permeability.^19^ Substrate mechanics also play a role in monocyte adhesion. For example, Mackay and Hammer observed increased monocyte attachment to hydrogels coated with E-selection as a function of stiffness, but they found no stiffness-dependence for gels coated with P-selectin,^20^ highlighting the complex interplay between biochemical and biophysical cues in extravasation. Moreover, stiffer substrates are required to properly recruit and stabilize ICAM-1 on endothelial cells.^21^ Collectively, these studies demonstrate that investigation of the key factors and molecular mediators that govern extravasation requires recapitulation of native perivascular matrix mechanics.

A variety of model systems have been developed to investigate the key molecular mechanisms that govern extravasation. Key chemokines secreted by monocytes, including interleukin-8 (IL-8) and monocyte chemoattractant protein-1 (MCP-1), which regulate firm monocyte adhesion and extravasation from the vascular endothelium, have been identified by molecular binding assays.^22,23^ Electrophoresis, microarrays, and polymerase chain reaction (PCR) techniques have allowed for the evaluation of monocyte and endothelial gene and protein expression that correlate with extravasation events in response to inflammatory factors. For example, lipopolysaccharides (LPS) induce E-selectin, VCAM-1, and ICAM-1 gene expressions,^24^ whereas interferon gamma (INF-γ) and interleukin-4 (IL-4) upregulate MCP-1 mRNA and protein expressions.^25,26^ In addition, electron microscopy has provided spatial resolution for key signaling pathways including IL-8, which, after being secreted by monocytes, is concentrated at the apical surface of endothelial cells prior to internalizations.^27^ Furthermore, the combination of these data with animal models and computational simulations has elucidated possible models of paracellular and transcellular migration of leukocytes across endothelial barriers.^28–30^ Despite enabling significant progress in identifying key governing pathways, these approaches have limitations, including a lack of physiological architecture and mechanics in *in vitro* systems,^31^ and divergent mechanisms in mouse and human models.^32,33^ For example, the commonly used mice strain C57B1/6 exhibits cell-mediated immunity and Natural Killer (NK) cell activity that is significantly higher than other mice strains,^34^ and which is not representative of humans.^35^

Microfluidic systems address some of these key limitations, particularly, the need for more physiologic, three-dimensional microenvironments *in vitro*.^36,37^ Recently, a number of microfluidic approaches have been developed for the study of extravasation,^38–40^ as recently reviewed Ma *et al*.^41^ However, the majority of these approaches are used to study cancer cell extravasation during metastasis, while the study of leukocytes and immune cell extravasation has received less attention. A few experimental approaches have been developed to study leukocyte extravasation in microfluidic devices, including endothelial cells cultured as a monolayer on a porous membrane,^32,42,43^ vascular networks embedded within collagen hydrogels,^39^ and endothelial monolayers attached to a rectangular PDMS channel.^44^ Studies using these platforms have contributed to a fundamental and critical understanding of the mechanisms of extravasation, such as the relation between cancer cell extravasation and the expression of adenosine receptors^39^ and late metastatic markers^44^ or the inability of leukocytes to extravasate when treated with pertussis toxin.^32^ However, there exist significant limitations in the physiological relevance of these approaches, including the lack of three-dimensional native architecture and/or physiologic substrate mechanics and hemodynamics.

Here, we present a microfluidic device and an approach that allows the formation of a continuous, lumenized, cylindrical monolayer of endothelial cells embedded within a collagen type-I matrix.^45^ The diameter of this vessel varies between 150 and 250 *μ*m, resembling physiological arterioles and venules.^46,47^ In vessels of this size, mechanotransduction of hemodynamic shear stresses facilitate interactions between immune and endothelial cells and immune cell extravasation.^35,48^ Using this platform, we demonstrate key aspects of physiological monocyte extravasation, including arrest to the apical vascular endothelial surface, crossing of the vascular endothelial barrier, and 3D migration through the hydrogel matrix. We further investigate the effect of hemodynamic shear stress and perivascular collagen concentration on extravasation, and the results of these studies demonstrate a critical role for biophysical stimuli in extravasation.

## METHODS

### Cell culture

Human Umbilical Vein Endothelial Cells (HUVECs) were grown in EGM-2 medium supplemented with 2% of Fetal Bovine Serum (FBS), hydrocortisone, Vascular Endothelial Growth Factor (VEGF), R3 Insulin-like Growth Factor 1 (R3 IGF-1), ascorbic acid, human Epidermal Growth Factor (hEGF), Gentamicin sulfate/Amphotericin (GA-1000), and heparin (medium EGM-2, Lonza, Basel, Switzerland). Cells were used from passage number 2 to 10, consistent with manufacturer recommendations to assure the viability and an adequate metabolism of cells.

THP-1 (ATCC^®^ TIB202™, Manassas, VA, USA) is a commercial cell line of monocytes isolated from peripheral blood. THP-1 was grown in suspension in RPMI-1640 media supplemented with l-glutamine, 2% of FBS and ampicillin/streptomycin (RPMI-1640, Gibco, Gaithersburg, MD, USA), and THP-1 were used from passage number 0 to 7.

### Fabrication of microfluidic devices

**FIG. 1.**
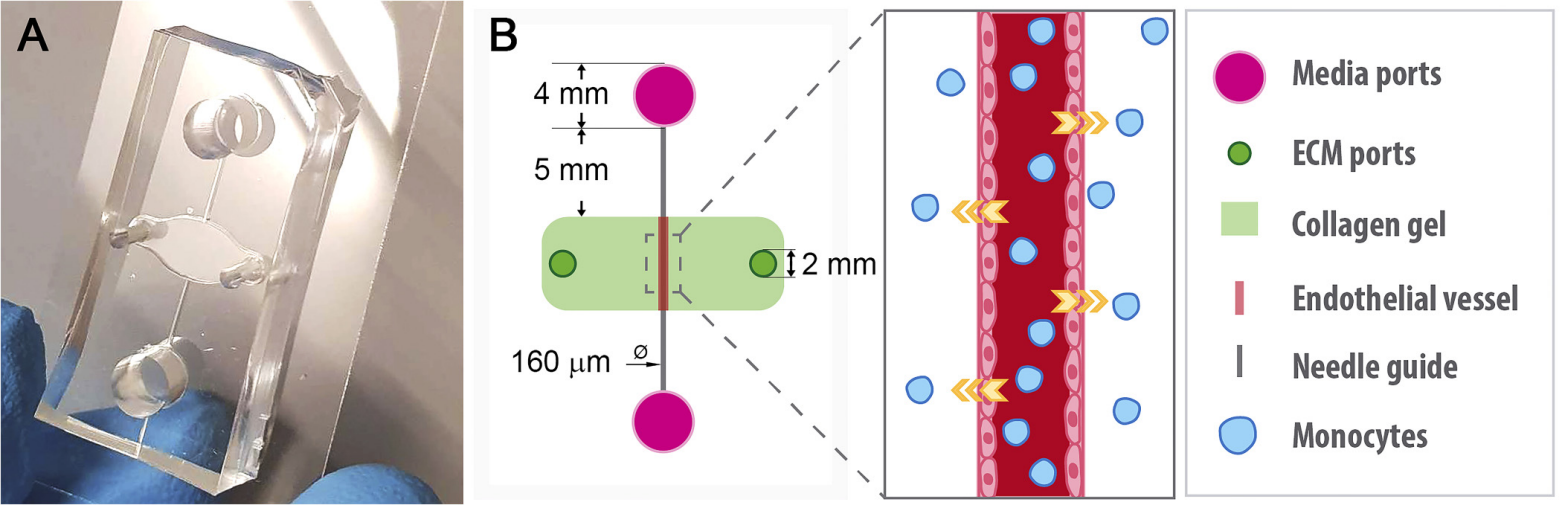
Microfluidic platform for investigating monocyte extravasation. (a) Photograph of device bonded to (24 × 40 mm^2^ coverslip). (b) Graphical representation of the device (not to scale) that consists of a central chamber with two ports (dark green) through which the collagen gel (light green) is introduced and polymerized. A channel formed by an acupuncture needle connects two reservoirs filled with the medium (bright pink) to the collagen gel region. HUVECs line the channel in the collagen gel (pink), and hydrostatic pressure gradients induced by the rocker induce flow through the channel. Monocytes (blue) are flowed through the vessel, and extravasation from the lumen into the collagen hydrogel is investigated using light microscopy.

Microfluidic devices (Fig. 1) were prepared following the protocol developed by Polacheck *et al.*,^16^ with minor modifications. Briefly, polydimethylsiloxane (PDMS) molds were generated by the mixture and degasification of the curing agent of Sylgard 184 silicone elastomer and the base in a 1:10 ratio, poured onto plastic molds replica molded from silicon master molds patterned by photolithography, and incubated at 60 °C for 24 h. Next, PDMS mold was separated from the plastic molds, and devices were individually cut and autoclaved. PDMS devices and glass coverslips (22 × 40 mm^2^ cover slips, Menzel-Gläser, Brunswick, Germany), pretreated with iso-2-propanol, were treated with oxygen plasma for 30 s to surface activate the PDMS and promote glass-PDMS bonding. Sealed devices were incubated at 100 °C for 10 min, then treated with 1 mg/ml poly-d-lysine (Sigma, St Louis, MO, USA) for one-channel devices or 0.01% w/v poly-l-lysine (Sigma, St Louis, MO, USA) for two-channel devices, for at least 1 h at room temperature. Then, washed with de-ionized water, treated with 1% glutaraldehyde for 15 min, and washed with de-ionized water for 24 h on a shaker.

### Formation of the endothelial vessel

After fabrication, bonding, and surface treatment, the devices were sterilized in a 70% ethanol solution for 30 min. A 0.16 mm diameter acupuncture needle (Φ 0.16 × 40 mm, Seirin, Shizuoka, Japan), pretreated with 0.01% bovine serum albumin (BSA) in phosphate-buffered saline (PBS) for 30 min, was inserted into the device and sterilized with ultraviolet light for 15 min. Subsequently, reconstituted type-I collagen derived from rat tail (Corning, Glendale, AZ, USA) was buffered to a pH of 7.5 with 1*N* NaOH in sterile H_2_O, 10× dPBS with phenol (Euroclone, Milan, Italy) and EGM-2 medium, and was introduced in the central region of the device. Two different collagen concentrations were tested, 2.5 and 6 mg/ml, starting from stock concentrations at 4.33 and 9.44 mg/ml, respectively. Devices were moved to a humidified incubator at 37 °C for at least 2 h, and device reservoirs were filled with EGM-2 media to avoid dehydration of the hydrogel. Next, the needle was removed, and the device was sealed with vacuum grease (Millipore Sigma, Saint Louis, MO, USA). For two-channel devices, the same procedure was performed with two needles inserted into each device.

Fresh EGM-2 was introduced into devices, and devices were moved to a laboratory rocker within a humidified incubator to wash devices for 24 h. The next day, a HUVEC suspension with a final concentration of 2 × 10^6^ cells/ml in EGM-2 was introduced into the device reservoirs, and cell adherence to the central channel in the collagen gel was observed by phase contrast microscopy (Nikon D-Eclipse C1 Confocal Microscope, 10× lens, Nikon Instruments, Tokyo, Japan). As previously described, when the cylindrical space was covered by adequate density of HUVECs,^16^ cell-containing media replaces with fresh media and devices were incubated at 37 °C for 24 h on a rocker at 30° and 5 cycles/min to introduce oscillatory, reciprocating flow through the vessel lumen.

### Extravasation assays

For the extravasation assays, a solution of 7.5 × 10^5^ cell/ml of THP-1 monocytes was resuspended in EGM-2 media and introduced into device ports. Devices were incubated at 37 °C for 24 h prior to analysis (Fig. 1). To study the influence of flow in the extravasation process, devices were pretreated with oscillating flow for 24 h before adding the monocytes, while static devices were maintained on a shelf of the same incubator for 24 h as well, prior to the addition of monocytes. To analyze the role of collagen density, extravasation assays were conducted with 2.5 and 6 mg/ml collagen hydrogels as described above.

### Inmunofluorescence

Devices were washed with PBS + 0.5 mM MgCl_2_ + 1 mM CaCl_2_ (PBS++), fixed with 4% paraformaldehyde (PFA) at 37 °C for 20 min on the oscillatory rocker, and washed again with PBS++. Samples were permeabilized with 0.1% Triton X (Calbiochem, Darmstadt, Germany) for 20 min and washed with PBS++. Subsequently, devices were rocked with 4′,6-diamino-2-fenilindol (DAPI) (1:1000) (Invitrogen, Carlsbad, CA, USA) and rhodamine phalloidin (1:200) (Thermo Fisher Scientific, Madrid, Spain) in 2% BSA in PBS++ at room temperature for 20 min. After washing with 2% BSA, devices were incubated with primary antibody against VE-cadherin (1:200) (Santa Cruz Biotechnology, Santa Cruz, CA, USA) in 2% BSA in PBS++ at 4 °C overnight. Next, anti-goat Alexa Fluor 647 secondary antibody (1:250) (Thermo Fisher Scientific, Madrid, Spain) in 2% BSA in PBS++ was added at room temperature for 2 h while protected from light. Finally, devices were washed with 2% BSA and stored at 4 °C. Maximum intensity projections were synthesized from Z-stack series obtained with a laser-scanning confocal microscopy (FV3000, Olympus) at 20× magnification (20× C Plan fluor 0.7 NA air objective, Olympus).

### Image acquisition and analysis

Data for extravasation assays were obtained from the analysis of fixed immunofluorescence images taken as described above with a 20× objective. The number of extravasated and adhered monocytes, the monocyte migration distance, and diameters were measured manually using Fiji.^50^ Monocytes were identified and differentiated from HUVECs by their small size, rounded shape, and different cell refractive index, which gave them a different shade of gray under the bright-field microscope.^51,52^

Alignment of phalloidin and VE-cadherin networks was determined by analyzing the maximum intensity projection of seven independent assays. After the projection, vessel images were split in square patches and their walls were removed to avoid bias in the main direction of the vessel (Fig. S1 in the supplementary material). The alignment index (α) was estimated using a discrete Fourier Transform (FT) method as described previously.^53,54^ This index ranges from 0 to 1, with 1 meaning a complete alignment of the network and 0 a random orientation.

### Permeability assays

Diffusive permeability of vessels was quantified as previously described.^16^ Briefly, EGM-2 supplemented with 70-kDa fluorescent dextran TexasRed (200 *μ*g/ml) (Sigma-Aldrich, Saint Louis, MO, USA) was introduced into the vessel, and images were taken at 10× magnification every 5 s for 50 cycles at the median transversal plane of the vessel. A total flux of dextran transported across the vascular wall was quantified by measuring the total intensity within the vessel for each time point (I_0_), and the total intensity outside of the vessel (I) as a function of time. The radius of the vessel (r), intensity within the vessel (I_0_), and rate of change of intensity outside of the vessel (δI/δt) were used to determine P_d_ through the following relation:

$$P_{d}=\left(\frac{2r}{I_{0}}\right)\left(\frac{\delta I}{\delta t}\right).$$ (1)

### Collagen hydrogel characterization

Collagen hydrogels at mass concentrations of 2.5 and 6 mg/ml were prepared as described above and frozen in liquid nitrogen overnight prior to lyophilization (ScanVac CoolSafe 110-4, Labogene, Lynge, Denmark) for 44 h. Samples were then deposited in holders on carbon tape and coated with a 14 nm layer of palladium to increase conductivity. The gels were visualized and photographed using a field scanning electron microscope (CSEM-FEG INSPECT F50, FEI Company, Hillboro, OR, USA) at resolutions of 10, 20, 50, and 100 K.

### Determination of hydraulic permeability

Following removal of needles in a two-channel device, all device reservoirs were emptied and filled with 70-kDa fluorescent dextran Fluorescein (FITC) (200 *μ*g/ml) (Sigma-Aldrich, Saint Louis, MO, USA) in PBS. These devices were then incubated overnight at 37 °C to allow the dextran to permeate the collagen hydrogel. The reservoirs were then emptied, and glass capillary tubes were inserted into the reservoirs of one channel. Any gaps between the PDMS and glass were sealed using vacuum grease, and the 70-kDa FITC solution was added to the glass reservoirs to apply a hydrostatic pressure of 5, 10, or 20 mmH_2_O. Fluorescence recovery after photobleaching (FRAP) was performed by finding the median transversal plane of the vessel and photobleaching a 50 pixel circle in the collagen gel between the two channels for 3 s, followed by continuous imaging of the bleached circle every second for 30 s. The velocity of the interstitial flow induced by the hydrostatic pressure was calculated using a custom Matlab code that fit a circle to the bleached region and determined circle displacement across the time series.

## RESULTS AND DISCUSSION

### Microfabricated blood vessels that mimic physiologic vasculature

**FIG. 2.**
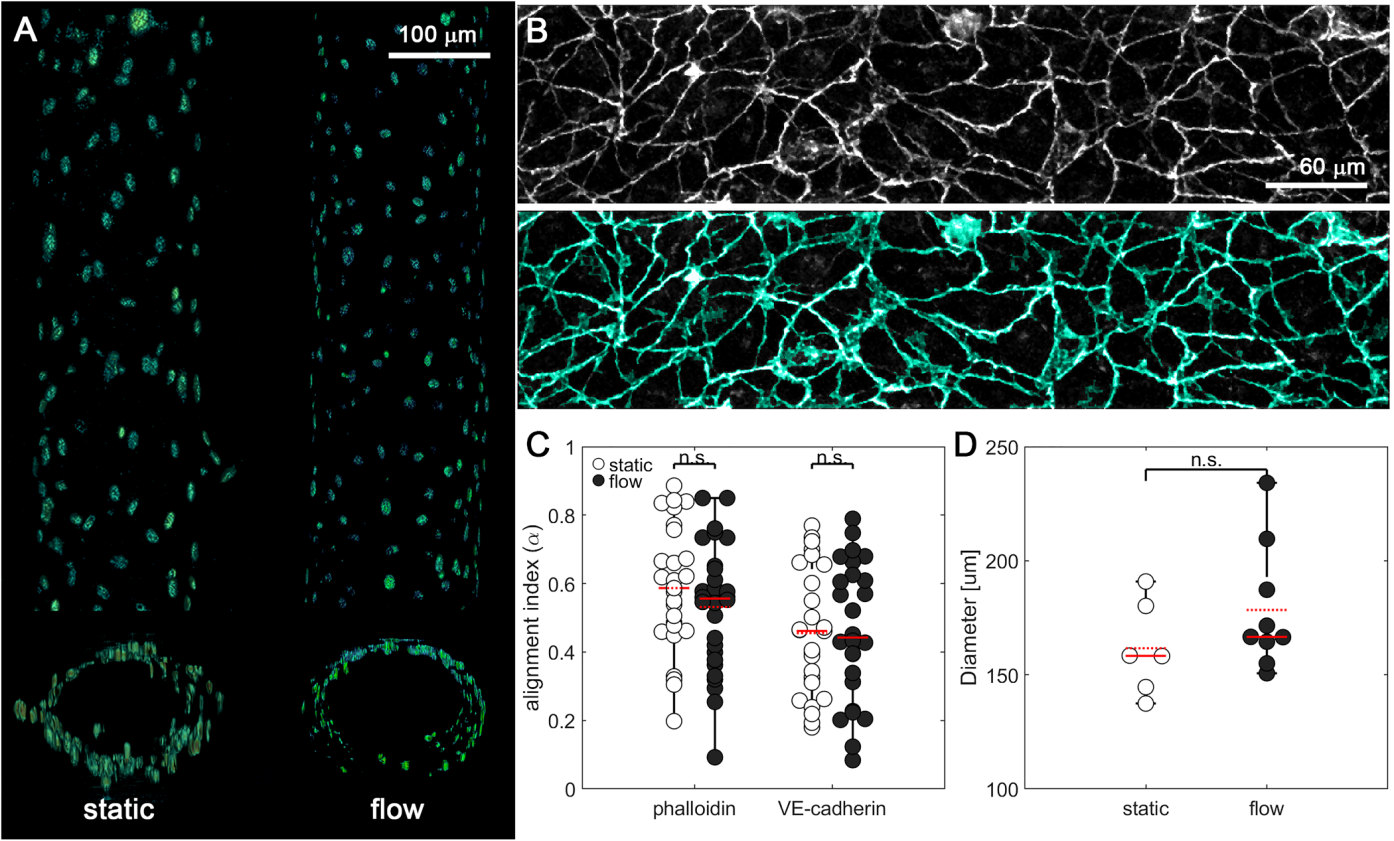
Endothelial vessels embedded in 2.5 and 6 mg/ml collagen gels within the microfluidic device. (a) Three-dimensional reconstruction from nuclei staining with DAPI, showing a longitudinal (upper) and a transverse (lower) view from a static and a flow vessel. (b) First row shows a gray scale example of the VE-cadherin staining of a vessel (maximum intensity projection), while second row shows, superimposed in cyan, the segmentation of the former performed by simple thresholding. (c) Phalloidin and VE-cadherin alignment index (alpha) of the maximum fluorescence projection of an endothelial vessel, with 1 being the complete alignment parallel to the vessel. (d) Diameters of each static and flow endothelial vessel. For all plots, each data point represents data from an individual device, and solid and dashed red lines represent the median and mean values, respectively. ANOVA tests were performed to determine statistical significance. *p < 0.05.

We fabricated a microfluidic cell culture system to develop microfabricated blood vessels, consisting of a perfusable channel lined with HUVECs embedded in 3D collagen hydrogel (Fig. 1). After seeding with endothelial cells, these devices were cultured with the oscillatory flow or under static conditions for 24 h. To characterize endothelial cell distribution within devices, we fixed the devices, stained them with DAPI, and performed a three-dimensional reconstruction of the spatial arrangement of the nuclei of endothelial cells forming the vessel [Fig. 2(a)]. These reconstructions reveal a continuous cylindrical endothelial monolayer embedded within the collagen type-I hydrogel. It has been shown previously that application of physiologic hemodynamic shear stress leads to alignment of the actin cytoskeleton.^55^ To measure the alignment of filamentous actin fibers, we stained fixed devices with rhodamine phalloidin and anti-VE-cadherin antibodies. VE-cadherin is an adherent junction protein that, in part, regulates endothelial permeability,^56^ and assembly of VE-cadherin-containing junctions indicates the establishment of endothelial barrier function.^57^

By performing a simple threshold segmentation of the VE-cadherin staining, we observed that all pixels were connected in a lattice of cell–cell junctions as a single object [Fig. 2(b)]. This suggests that there are no gaps between the HUVECs forming the vessel and that it is in fact composed of a continuous monolayer of endothelial cells. We then computed an alignment index using a Fourier transform analysis of max intensity projections from z-stacks acquired with confocal microscopy. With this approach, we observed that the cytoskeleton presents an alignment index slightly greater than 0.5 and that VE-cadherin alignment was approximately 0.5. In both cases, there were no significant differences between vessels cultured under static and flow conditions [Fig. 2(c)]. This index value indicates that the fibers are aligned mostly parallel to the vessel, in the same direction as flow. We further used devices stained with phalloidin to measure vessel diameter, which ranged from 125 to 250 *μ*m [Fig. 2(d)] and is comparable to the diameter of human arterioles and venules.^46,47^

### Fluid shear stress increases monocytes adherence to the vessel wall but decreases extravasation

Physiologically, fluid shear stresses stimulate monocytes and endothelial cells by increasing their expression of adhesion proteins, including E-selectin and ICAM-1, and by reorganizing the cytoskeleton of endothelial cells.^12,43^ To investigate the role of endothelial response to hemodynamic shear stress on monocyte attachment and extravasation, endothelial vessels were pretreated with 24 h of oscillatory flow or were cultured in static conditions. After this differential treatment, monocytes were added to one of the reservoirs to introduce a hydrostatic pressure gradient resulting in the flow of monocytes through the endothelial lumen for 24 h.

**FIG. 3.**
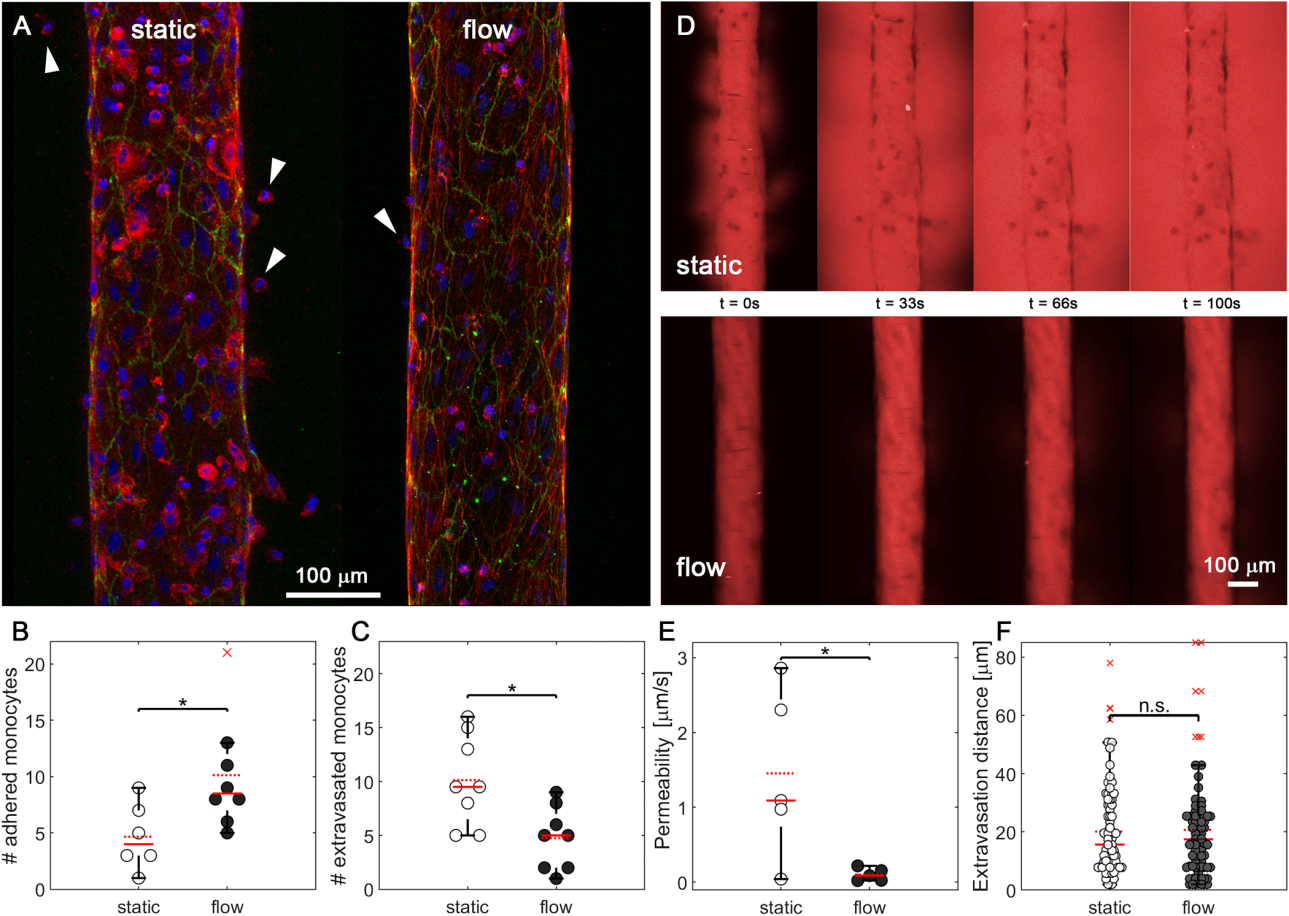
Effect of the fluid flow stimulation on extravasation. (a) Endothelial vessels in 2.5 mg/ml hydrogels in static (left) and flow-pretreated (right) conditions. Vessels are stained for actin (red), VE-cadherin (green), and nucleus (blue). Extravasated monocytes are indicated with white arrows. (b) Number of monocytes adhered to the endothelial lumen in static (white) and flow-pretreated (black) vessels. (c) Number of monocytes extravasated from the lumen to the surrounding hydrogel in static (white) and flow-pretreated (black) vessels. (d) Permeability assay with 70 kDa fluorescent dextran (red) added to the endothelial lumen. Images are single confocal slices taken after the addition of dextran in a static (upper) and a flow-pretreated (lower) vessel. (e) Permeability of static (white) and flow-pretreated (black) vessels. (f) Distance migrated by monocytes from the endothelial wall to the collagen gel in static (white) and flow-pretreated (black) vessels. For all plots, each data point represents data from an individual device, and solid and dashed red lines represent the median and mean values, respectively. Red crosses represent outliers. ANOVA tests were performed to determine statistical significance. *p < 0.05.

Treatment of vessels with flow resulted in an increase in adherent monocytes and a decrease in extravasated monocytes [Fig. 3(a)]. To quantify this observation, monocyte nuclei stained with DAPI were counted in image stacks acquired with a laser-scanning confocal. Vessels pretreated with flow demonstrated between 5 and 20 adherent leukocytes per vessel (counted along the entire vessel), while static vessels did not exceed 10 monocytes per vessel [Fig. 3(b)]. This statistically significant effect of flow is supported by previous work demonstrating that flow increases the expression of proteins involved in monocyte adherence, including E-selectin and ICAM-1, in vascular endothelial cells.^12^ Despite the increase in monocyte adherence to the lumen of flow-stimulated vessels, we found a decrease in the number of monocytes that traversed the endothelial barrier and migrated into the collagen hydrogel. As quantified from confocal z-stacks, extravasated monocytes in static vessels ranged from 5 to 20 per vessel, whereas when treated with the flow, the range is reduced to 1 to 10 [Fig. 3(c)]. We hypothesized that the decreased rate of extravasation could be due to the effects of flow on vascular endothelial barrier integrity, as permeability of endothelial cell monolayers decreases with the applied flow.^43^

To test this hypothesis and to determine whether flow impacts barrier function of the vascular endothelial monolayer, we quantified the diffusive flux of fluorescently tagged 70 kDa dextran from the vessel lumen into the hydrogel using time lapse confocal microscopy. After 100 s of dextran perfusion, we observed a stark difference between the distribution of dextran throughout the hydrogels, with the levels of dextran within the subluminal matrix nearly equivalent to those in the lumen for vessels cultured in static conditions [Fig. 3(d)]. To quantify this observation, we measured the diffusive permeability of vessels, a measure of the barrier to diffusive flux in response to discrete changes in concentration across a membrane, in this case, the endothelial monolayer. We found a significant difference in the diffusive permeability of vessels cultured in static vs flow conditions, with the mean permeability more than ten times higher in static vessels (mean = 1.45 *μ*m/s) than in flow-treated vessels (mean = 0.10 *μ*m/s) [Fig. 3(e)], consistent with previous observations.^58^ Previous work has demonstrated that a flow-mediated reduction in vascular permeability is driven by the assembly of VE-cadherin-containing adherens junction complexes,^17^ and we hypothesize that this junctional assembly presents a barrier to monocyte migration and extravasation. This hypothesis is further supported by work demonstrating that VE-cadherin expression levels are increased with flow^59^ and is an essential part of a mechanosensory complex that plays a role in the establishment and maturation of tight junctions,^60,61^ and that mature adherens junctions present a barrier to monocyte extravasation.^3^

Interestingly, once monocytes have crossed the endothelial barrier, there is no difference in the total migration distance through the collagen gel. In both conditions, monocytes travel around 20 *μ*m from the vessel wall [Fig. 3(f)]. These results are similar to previously published data by Boussommier-Calleja *et al.*, which indicated that after extravasation, monocytes tended to migrate between 10 and 20 *μ*m.^62^ Our longer migration distances might be explained by differences in the hydrogels used. Boussommier-Calleja worked with 3 mg/ml fibrin collagens, whereas we use 2.5 mg/ml collagen gels. In addition, fibrin gels present different fiber organization, with smaller pore sizes.^63^ It has also been demonstrated that cells encounter greater steric hindrance in their advance and migrate shorter distances when traversing fibrin gels compared to collagen gels.^64^

### Increased collagen matrix concentration decreases vascular barrier integrity and increases extravasation

**FIG. 4.**
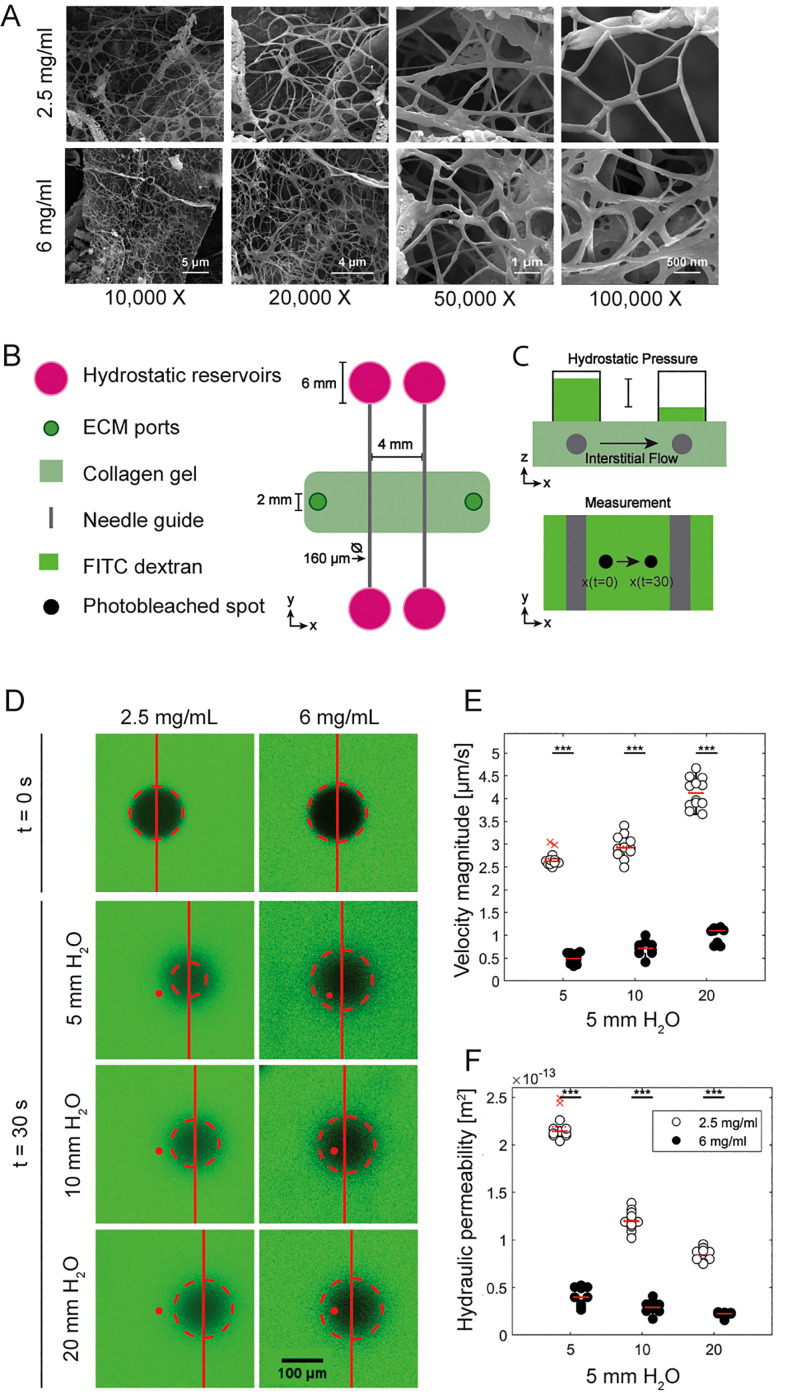
3D structure and hydraulic permeability of 2.5 and 6 mg/ml collagen gels. (a) Scanning electron microscope (SEM) images of 2.5 and 6 mg/ml gels at resolutions of 10, 20, 50, and 100 K. (b) Diagram of two-channel device used to measure hydraulic permeability. Reservoirs are connected to the media ports to allow application of a defined pressure gradient across the collagen hydrogel. (c) The experimental setup of fluorescence recovery after photobleaching (FRAP) method used to measure the hydraulic permeability. A known pressure gradient is applied to hydrogels immersed in the dextran-containing medium, and the velocity magnitude of a photobleached circle is measured with timelapse fluorescence microscopy. (d) Sample FRAP data demonstrating spot displacement in response to an applied hydrostatic pressure gradient. (e) Measured velocity of fluid flow between two channels. (f) Hydraulic permeability of collagen hydrogels as a function of applied pressure gradient calculated from Darcy's law. For all plots, each data point represents data from an individual device, and solid and dashed red lines represent the median and mean values, respectively. ANOVA tests were performed to determine statistical significance. ***p < 0.001.

The physical properties of the endothelial basement membrane and intimal tissue are known to modulate vascular barrier function and inflammation.^65^ Previous work demonstrated that increased intimal stiffness results in increased vascular permeability and immune cell extravasation through activation of Rho-mediated contractility in endothelial cells.^66^ To elucidate the role of collagen matrix concentration on permeability and extravasation in our microfluidic model, we synthesized vessels in 2.5 and 6 mg/ml collagen type-I hydrogels. It has been previously demonstrated that a change in collagen concentration impacts the biophysical properties of hydrogels. Our group previously analyzed the storage shear modulus (G′), which indicates the elastic response of a material to shear stress, as a function of collagen mass concentration. We found that 2.5 mg/ml hydrogels were characterized by a G′ of 62.14 ± 4.87 Pa, while 6 mg/ml hydrogels were characterized by a G′ of 254.05 ± 29.06 Pa (Fig. S2 in the supplemental material).^67^ Oliveros *et al.* performed a computational spatial characterization of the collagen fibers that comprise the solid phase of the hydrogel, and determined that increasing collagen concentration resulted in a decrease in pore size and porosity of the material. In addition, they found that increasing collagen concentration resulted in an increase in the number of fibers, while the length and radius of the fibers decreased.^68^ We examined the structure of collagen hydrogels with SEM [Fig. 4(a)]. Consistent with previous results, at lower magnifications, we found that the 2.5 mg/ml hydrogels by increased void space when compared to the 6 mg/ml hydrogels and increased suggests the 6 mg/ml gels are comprised of thicker fibers with more junctions between fibers [Fig. 4(a)]. Together these data indicate that the increase in collagen concentration results in changes in the spatial distribution of collagen fibers, which we expect to impact mechanical properties. To determine whether these structural differences presented differential functional barriers to migration, we determined the hydraulic permeability, which is related to the effective pore size. The hydraulic permeability of the different collagen compositions was determined by applying a hydrostatic pressure of 5, 10, or 20 mmH_2_O using 70-kDa FITC dextran across a two-channel device and measuring the resulting velocity across the collagen between the two channels [Fig. 4(b)]. FRAP was performed by photobleaching a spot between the channels and imaging the displacement of the circle over time [Fig. 4(c)]. This displacement was used to calculate the velocity of the fluid [Fig. 4(d)] due to the applied hydrostatic pressures and used to calculate the hydraulic permeability in each condition, and statistically significant decreases in hydraulic permeability were found between the two hydrogel compositions [Fig. 4(e)].

**FIG. 5.**
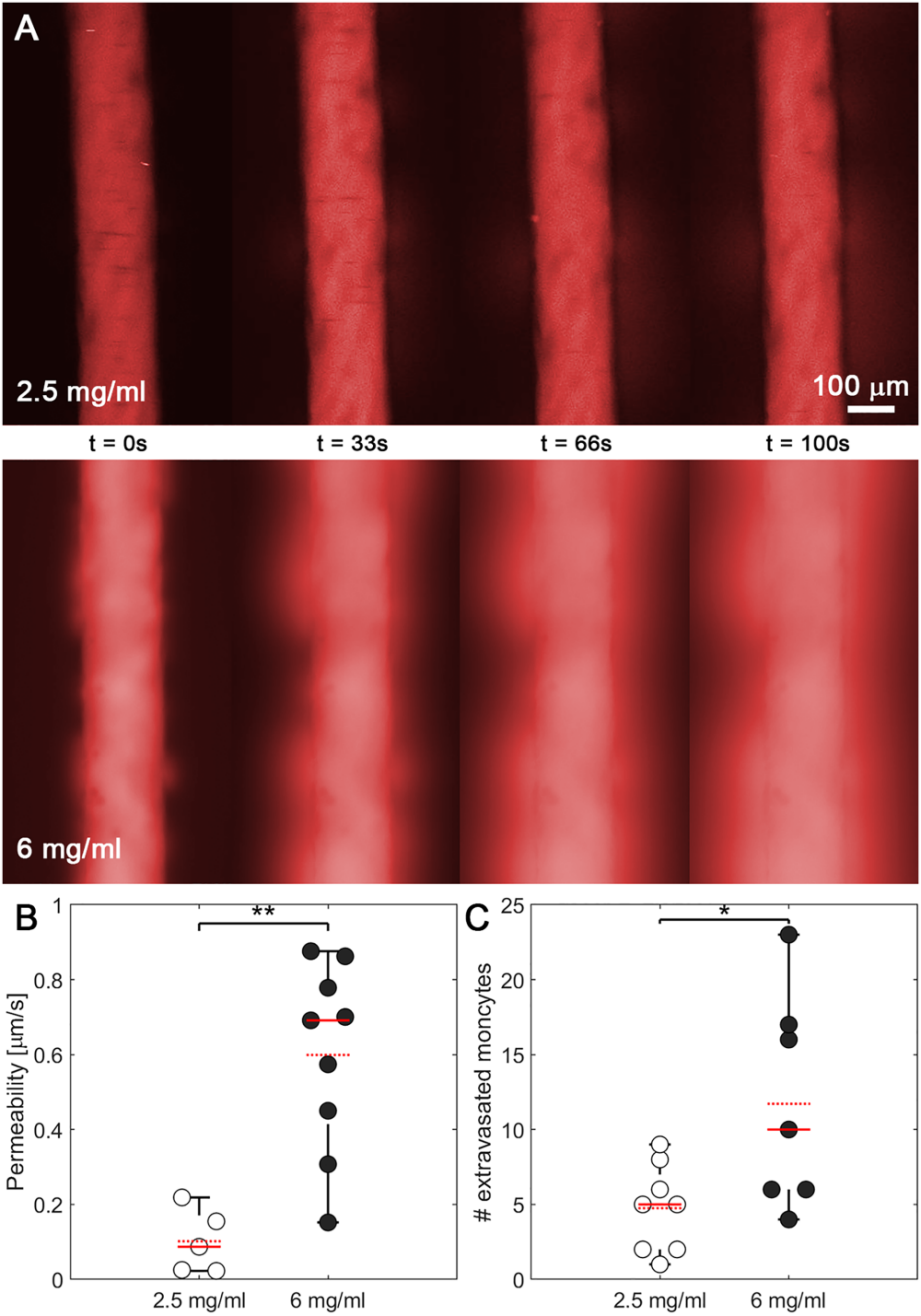
Effect of the collagen concentration on vascular permeability and monocyte extravasation. (a) Diffusive permeability assay with fluorescent dextran (red) diffusing from the vessel lumen through the endothelial monolayer at different times (t = 0, 33, 66, and 100 s) in vessels formed in 2.5 (upper) and 6 mg/ml collagen hydrogels (lower). (b) Diffusive permeability of vessels embedded in 2.5 (white) and 6 mg/ml collagen hydrogels (black). (c) Number of monocytes extravasated from the lumen into the surrounding hydrogel in 2.5 (white) and 6 mg/ml collagen hydrogels (black). For all plots, each data point represents data from an individual device, and solid and dashed red lines represent the median and mean values, respectively. ANOVA tests were performed to determine statistical significance. **p < 0.01; *p < 0.05.

To determine the impact of collagen concentration on vascular barrier function and monocyte extravasation, we repeated the diffusive permeability assay in vessels synthesized in 2.5 mg/ml vs 6 mg/ml collagen hydrogels and exposed to flow. Increased collagen concentration resulted in significant increases in vascular permeability [Fig. 5(a)]. Quantitatively, the average permeability in high collagen concentration gels (mean = 0.60 *μ*m/s) is six times higher than the mean obtained in lower collagen concentration gels (mean = 0.10 *μ*m/s). In 2.5 mg/ml gels, the permeability ranges from 0 to 0.2 *μ*m/s, while in 6 mg/ml gels, it ranges from 0.1 to almost 0.9 *μ*m/s [Fig. 5(b)]. Interestingly, despite the decreased pore size of the 6 mg/ml collagen gels, there were nearly twice as many extravasated monocytes in the 6 mg/ml hydrogels compared to the 2.5 mg/ml hydrogels. [Figure 5(c)]. Our data suggest that the integrity of endothelial vessels plays an essential role in monocyte extravasation and is more impactful than the steric hindrance caused by the higher collagen concentrations.

## CONCLUSIONS

Here, we introduce a microfluidic model of a monocyte-laden perfusable 3D blood vessel and demonstrate the ability to recapitulate monocyte arrest, extravasation, and 3D migration through a subluminal matrix. Importantly, this model system improves upon traditional assays by allowing for modulation of key hemodynamic parameters, including pressure and flow, for vessels embedded within a three-dimensional extracellular matrix with varying density. We demonstrate that both flow and matrix density play key roles in adhesion, extravasation, and migration, and, interestingly, our data demonstrate a complex interplay among biophysical parameters that govern monocyte–endothelial interactions. While flow promotes the adhesion of monocytes to the vascular wall, the number of cells that extravasate into the subluminal matrix decreases with the flow, suggesting that extravasation, rather than adhesion, is the rate limiting step for immune cell trafficking in the presence of flow. This idea is supported by vascular permeability data, which demonstrates increased barrier to diffusion of 70 kDa dextran in the presence of flow and further suggests that targeting vascular permeability could be an effective strategy for screening the passage of molecules or other cells through the endothelial wall, such as drugs that must diffuse to their target organ or pathogen, or metastatic cells in cancer progression.

Devices fabricated in a similar manner, by casting a hydrogel around a needle to ultimately form a perfusable vessel embedded within a 3D hydrogel, have been used for numerous applications to study the impact of the biochemical and biophysical microenvironment on microvascular morphogenesis and function. Such devices have been implemented to screen the effects of pro-angiogenic cocktails on neovascularization,^69^ to define the role of matrix degradability in angiogenesis,^70^ and to determine the role of inflammatory factors in governing lymphatic drainage.^71^ Yet, in most of these studies (reviewed in Ref. 72), cell culture media is used as a blood surrogate, and the contributions of circulating cells toward microvessel function are not considered, despite increasing evidence that leukocytes and immune cells play critical roles in microvascular development, homeostasis, and dysfunction.^73^ Our results demonstrate that such platforms are compatible with circulating cells and suggest that mechanistic studies enabled by these devices could allow for dissection of key signals involved in pathologies such as fibrosis and cancer, where changes in ECM composition and mechanics occur concomitantly with changes in hemodynamics, to identify and screen therapeutic interventions. One challenge in the use of the current device for drug screening or other applications requiring a large number of devices is the overall time required to fabricate and conduct studies in the device proposed here (>72 h from pouring PDMS to introducing monocytes). Much of this time (48 h) is spent washing the device to prevent glutaraldehyde-induced cytotoxicity. Recently, dopamine hydrochloride has been demonstrated as an alternative surface coating to glutaraldehyde that is much less cytotoxic and does not require such substantial washing.^49^ The use of dopamine hydrochloride could remove 48 h from our fabrication protocol and reduce the device assembly process to something that can easily be completed within 1 day.

While the application of flow impacted the rate of monocyte adherence and extravasation, the migration distance of monocytes into the collagen hydrogel was not dependent on the application of flow. Given that tissues demonstrate varying degrees of infiltration by monocytes and other circulating cells *in vivo*, it is likely that the platform described here does not recapitulate sufficient complexity to investigate mechanisms that lead to differences in cell migration behaviors beyond extravasation. By focusing only on the adhesion and extravasation process, we do not recapitulate some key cellular events that occur before and after the extravasation and that may influence the final outcome of monocyte or immune cell surveillance. The addition of chemical and biological stimuli from perivascular tissue, such as chemokines or molecules derived from microorganisms, would allow the study of the recruitment of monocytes to damaged tissue^8^ and subsequent differentiation into macrophages in the extracellular matrix.^74,75^ In this way, a more complete and physiologic model could be achieved. The introduction of perivascular signals and gradients will be the focus of future studies, as the lateral ports of the device described here (Fig. 1) provide access to the hydrogel tissue region and can be used to establish molecular or pressure gradients. Furthermore, recent work introducing epithelial ducts into lumenized vascular devices^76^ and coculturing diverse bacterial colonies on chip^77^ together illustrate the potential to recapitulate complex inflammatory responses *in vitro* through the integration of these approaches.

## SUPPLEMENTARY MATERIAL

See the supplementary material for details on computational image processing, hydrogel stiffness data, and analysis of correlations between vessel properties and monocyte extravasation.

## AUTHORS' CONTRIBUTIONS

S.P.-R. and W.J.P. conceived of the experiments. S.P.-R. conducted experiments involving monocytes and measures of vascular phenotype and permeability, and S.A.H. conducted hydraulic permeability measurements. S.P.-R., S.A.H., and C.B. processed data and prepared figures. S.P.-R., S.A.H., and C.B. wrote the manuscript, which was edited by J.M.G.-A. and W.J.P.

## Data Availability

The data that support the findings of this study are available from the corresponding author upon reasonable request.
